# Distinct adaptive strategies to cisplatin, vinblastine and gemcitabine in a panel of chemoresistant bladder cancer cell lines

**DOI:** 10.20517/cdr.2025.95

**Published:** 2025-09-12

**Authors:** Monika Cuprych-Belter, Agnieszka Łupicka-Słowik, Artur Anisiewicz, Martin Michaelis, Jindrich Cinatl Jr., Mateusz Psurski

**Affiliations:** ^1^Department of Experimental Oncology, Hirszfeld Institute of Immunology and Experimental Therapy, Polish Academy of Sciences, Wroclaw 53-114, Poland.; ^2^Faculty of Chemistry, Department of Organic and Medicinal Chemistry, Wroclaw University of Science and Technology, Wroclaw 50-370, Poland.; ^3^School of Natural Sciences, University of Kent, Canterbury CT2 7NZ, UK.; ^4^Dr Petra Joh Research Institute, Frankfurter Stiftung für krebskranke Kinder, Frankfurt am Main 60528, Germany.

**Keywords:** Transitional cell carcinoma, drug resistance, *in vitro* urinary bladder cancer model

## Abstract

**Aim:** Urinary bladder cancer (UBC) often develops chemoresistance, reducing treatment effectiveness. This study aimed to investigate diverse molecular mechanisms underlying acquired resistance by establishing and characterizing a comprehensive panel of UBC cell lines resistant to common chemotherapeutics.

**Methods:** Fifteen UBC cell lines were examined: three parental lines (RT-112, TCC-SUP, UMUC-3) and twelve derived sublines adapted to cisplatin, vinblastine, or gemcitabine. Drug sensitivity was assessed using the SRB assay. Resistance mechanisms were explored via quantitative real-time PCR (targeting genes including *ABCB1*, *dCK*, *hENT1*, *ECHDC1*, *TUBB3*), Western blotting (assessing proteins such as p21, Cyclin B, and Mcl-1), and biochemical assessment of glutathione levels and redox state.

**Results:** The adapted sublines exhibited distinct resistance profiles and cross-resistance patterns. Gene expression and protein analyses revealed drug- and lineage-specific alterations, involving factors such as p21, Cyclin B, and Mcl-1. Changes in glutathione metabolism were also associated with resistance. Notably, no single, universal mechanism accounted for resistance across the entire panel.

**Conclusion:** UBC cells develop diverse, context-dependent adaptive strategies to resist cisplatin, vinblastine, and gemcitabine. These findings highlight the complexity of chemoresistance mechanisms. The characterized cell line panel represents a valuable resource for future studies aimed at understanding and overcoming drug resistance in bladder cancer, suggesting that personalized therapeutic approaches may be necessary.

## INTRODUCTION

Urinary bladder cancer (UBC) is the tenth most common malignant disease and the thirteenth leading cause of cancer-related deaths worldwide. Men are more often affected than women, ranking sixth among the most frequently diagnosed cancers in men. In 2020, an estimated 573,000 new cases of bladder cancer were diagnosed, with 213,000 deaths recorded globally^[1]^. Most bladder cancers originate from the transitional (urothelial) epithelium lining the inner bladder wall, which is in direct contact with urine. This explains why tobacco smoking (responsible for half of all cases^[2]^), as well as exposure to contaminated water and toxic industrial chemicals (e.g., in textiles, paints, and rubber), are the major risk factors for transitional cell carcinomas (TCCs). Because these carcinogens accumulate gradually, bladder cancer incidence is strongly age-related^[3]^.

Bladder cancer is currently treated with several well-established, disease-stage-dependent regimens. Non-muscle-invasive cancers are typically managed with transurethral resection followed by intravesical Bacillus Calmette-Guérin vaccine (BCG) immunotherapy, complemented with intravesical chemotherapy (e.g., doxorubicin). Muscle-invasive cancers are usually treated with either the methotrexate, vinblastine, doxorubicin, and cisplatin (MVAC) regimen or the gemcitabine and cisplatin (GC) regimen, both of which yield satisfactory results. However, a significant number of patients experience disease relapse and eventually die due to tumor metastasis. Moreover, TCCs are generally recognized as chemoresistant, and failure of first-line therapy often leads to even more refractory, life-threatening malignancies that remain difficult to treat, even with newer drugs (e.g., vinflunine)^[4,5]^.

Drug resistance is a well-documented phenomenon in which cancer cells gradually adapt to therapy. Resistance - whether to chemotherapy or radiotherapy - is one of the major reasons treatments fail. Some histological cancer types, including many UBCs^[6]^, exhibit intrinsic resistance, whereas in most cases, resistance develops in response to treatment. The mechanisms involved include increased drug efflux and/or inactivation, DNA mutations, and metabolic reprogramming that alters drug targets and enhances DNA repair^[7]^.

Studying the development of chemoresistance is hampered by the scarcity of paired patient samples collected before and after therapy. Since large-scale databases such as TCGA also lack such longitudinal data (if any, they contain healthy-cancerous paired samples)^[8]^, research in this area remains heavily dependent on *in vitro* cell line models. While many studies focus on single drug-cell line pairs, there is a notable lack of comprehensive research directly comparing how different bladder cancer cell lines adapt to multiple drugs, thus obscuring the diverse strategies underlying acquired resistance. To overcome this gap, we developed and characterized a set of isogenic cell lines with acquired resistance to cisplatin (CDDP), gemcitabine (Gem), or vinblastine (VBL). These twelve models were derived from three distinct parental lines, namely RT-112, TCC-SUP, and UMUC-3, selected from 33 urinary bladder cell lines available in the Cancer Cell Line Encyclopedia (CCLE) to represent as diverse as possible clinical and molecular features of UBC [Table 1].

**Table 1:** Summary of the basic features of the parental cell lines used in the current study

**Feature^[8,9]^**	**RT-112**	**TCC-SUP**	**UMUC-3**
Tumor grade	Intermediate (Grade II)	High (Grade IV)	High (Grade III)
Invasiveness	Non-muscle invasive (NMIBC)	High-grade TCC	Muscle-invasive (MIBC)
Driver mutation	*FGFR3-TACC3* (fusion)	*PIK3CA* (activating E545K)	*KRAS* (activating G12C)
TP53 (p53) status	Mutated (R248Q; S183Ter; loss-of-heterozygosity)	Mutated (E349Ter; loss-of-heterozygosity)	Mutated (F113C; copy number neutral)
CDKN1A (p21) status	Homozygous deletion (no protein)	Wild-type (inducible)	Wild-type (impaired induction)
Genomic stability	Stable (near-diploid)	Moderately unstable	Highly unstable (aneuploid)
Sensitivity to CDDP, Gem, VBL^*^	CDDP: low; Gem: moderate; VBL: high	CDDP: high; Gem: moderate; VBL: low	CDDP: high; Gem: high; VBL: moderate

^*^Genomics of Drug Sensitivity in Cancer Database (GDSC1) accessed on 07/30/2025. TCC: Transitional cell carcinoma; CDDP: cisplatin; Gem: gemcitabine; VBL: vinblastine.

## METHODS

### Cell lines and culture conditions

The RT-112, TCC-SUP, and UMUC-3 cell lines were purchased from the American Type Culture Collection (ATCC; Rockville, USA). Drug-adapted sublines - RT-112^CDDP^, RT-112^Gem^, RT-112^VBL^, RT-112^CDDP/Gem^, TCC-SUP^CDDP^, TCC-SUP^Gem^, TCC-SUP^VBL^, and TCC-SUP^CDDP/Gem^ - were established by continuous exposure to stepwise-increasing concentrations of the respective cytostatics and are derived from the Resistant Cancer Cell Line (RCCL) collection^[10]^. The UMUC-3^CDDP^, UMUC-3^Gem^, UMUC-3^VBL^, and UMUC-3^CDDP/Gem^ cell lines were established at the Hirszfeld Institute of Immunology and Experimental Therapy of the Polish Academy of Sciences (HIIET, PAS), Wroclaw, Poland, using a similar approach.

All cell lines were cultured in Dulbecco’s Modified Eagle Medium (DMEM; Life Technologies, Scotland) supplemented with 10% (*v/v*) fetal bovine serum (FBS; GE Healthcare HyClone, Logan, USA), 2 mM L-glutamine (Sigma-Aldrich, Poznań, Poland), and antibiotics - 100 μg/mL streptomycin (Polfa Tarchomin, Warsaw, Poland) and 100 U/mL penicillin (Sigma-Aldrich, Poznań, Poland). Drug-resistant sublines were cultured in media containing the corresponding cytostatics: 8.3 µM cisplatin for UMUC-3^CDDP^ and UMUC-3^CDDP/Gem^, and 3.32 µM for RT-112^CDDP^ and TCC-SUP^CDDP^; 5 nM vinblastine for UMUC-3^VBL^ and 20 nM for RT-112^VBL^ and TCC-SUP^VBL^; 500 nM gemcitabine for UMUC-3^Gem^ and UMUC-3^CDDP/Gem^, and 75 nM for RT-112^Gem^, RT-112^CDDP/Gem^, TCC-SUP^Gem^, and TCC-SUP^CDDP/Gem^. One passage before experiments, cells were allowed to grow in a drug-free culture medium.

Mycoplasma contamination was tested using the Venor GeM Classic kit (Minerva Biolabs, Berlin, Germany), with negative results in all cases. Cells were cultured in a humidified atmosphere at 37 °C with 5% (*v/v*) CO_2_ and passaged twice weekly using ethylenediaminetetraacetic acid (EDTA)-trypsin solution (pH 8; HIIET, Wroclaw, Poland) as the detachment agent.

### Drugs

Bortezomib, combretastatin A-4, and vinflunine tartrate were purchased from Selleckchem (Munich, Germany) and stored at 80 °C as 50 mM solutions in dimethyl sulfoxide (DMSO; Avantor Performance Materials, Gliwice, Poland). Camptothecin, colchicine, gemcitabine hydrochloride, imatinib, trichostatin A, vinblastine sulfate, and vinorelbine tartrate were purchased from SigmaAldrich (Poznań, Poland) and stored under the same conditions. Cisplatin was purchased from Ebewe (Unterach am Attersee, Austria) and was maintained as a ready-to-use stock solution (1 mg/mL) stored at room temperature. Doxorubicin hydrochloride was purchased from Accord Healthcare Polska (Warsaw, Poland) and stored at +4 °C as a ready-to-use stock solution (2 mg/mL). Paclitaxel was obtained from Cytoskeleton Inc. (Denver, USA) and stored at 80 °C as a 2 mM solution in DMSO.

### Antiproliferative activity assessment by sulforhodamine B assay

Cells were seeded into 384-well plates (Greiner BioOne, Kremsmünster, Austria) at a density of 1 × 10^3^ cells/well. After overnight attachment, cells were exposed to test compounds at various concentrations (at least eight concentrations). After 72 h, the sulforhodamine B (SRB) assay was performed according to Skehan *et al.*, with minor modifications^[11]^. Briefly, 50 µL of culture medium was replaced with 30 µL/well of 20% (*w/v*) trichloroacetic acid (Avantor Performance Materials, Gliwice, Poland). After 1 h of incubation, plates were washed several times with tap water, followed by staining with 20 µL of 0.1% (*w/v*) SRB (Sigma-Aldrich, Poznań, Poland) in 1% (*v/v*) acetic acid (Avantor Performance Materials, Gliwice, Poland). After 30 min incubation at room temperature, unbound dye was removed with 1% (*v/v*) acetic acid, and bound dye was solubilized with 70 µL of 10 mM unbuffered tris(hydroxymethyl)aminomethane (TRIS; Avantor Performance Materials, Gliwice, Poland). The procedure was carried out using a Biotek EL-406 washing station (BioTek Instruments, USA). Absorbance was measured at 540 nm using a Biotek Hybrid H4 reader (BioTek Instruments, USA). Proliferation inhibition (%Inh) was calculated using the following formula:

**Figure:**
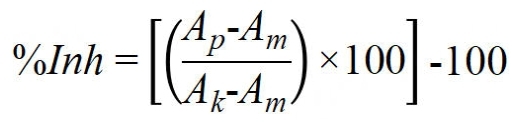


where *A_m_* is the absorbance of cell-free medium (blank), *A_k_* is the absorbance of vehicle-treated cells (control), and *A_p_* is the absorbance of compound-treated cells. IC_50_ (95%CI) were determined using GraphPad Prism 7.03 (GraphPad Software Inc., USA) by fitting a four-parameter variable slope equation ([inhibitor] *vs.* response). All values were based on at least three independent experiments.

For each cell line and compound, the resistance index (RI) was calculated as the IC_50_ ratio of resistant versus parental cell lines. RI values are reported directly as positive values when resistance was observed, and as negative values calculated as -(1/RI) when sensitization occurred.

### Selected drug resistancerelated gene expression analysis by real-time PCR

Total RNA was isolated using TRI Reagent (Molecular Research Center, Cincinnati, USA) according to the manufacturer’s instructions (based on the method developed by Chomczynski and Sacchi^[12]^). RNA quantity and purity were determined spectrophotometrically at 260 nm using a NanoDrop 2000 spectrophotometer (Thermo Fisher Scientific, Waltham, USA). RNA samples were enriched with RNAse inhibitors (EURx, Gdansk, Poland), treated with DNase I (Thermo Fisher Scientific, Waltham, USA) to remove genomic DNA, and reverse-transcribed into cDNA using iScript reverse transcriptase (Bio-Rad, Hercules, USA).

Real-time PCR was performed using TaqMan chemistry (Thermo Fisher Scientific, Waltham, USA). For each reaction, 25 ng of cDNA was used in technical triplicate, and four independent biological repeats were performed. Amplification consisted of 40 cycles of 95 °C for 15 s and 60 °C for 1 min, conducted on a ViiA^TM^ 7 Real-Time PCR System (Thermo Fisher Scientific, Waltham, USA). The following TaqMan probes were used: *TUBβ3* (Hs00801390_s1); *hENT1* (Hs01085706_m1); *SLC31A1* (solute carrier family 31 member 1; Hs00741015_m1); *MT2A* (metallothionein-2; Hs02379661); *ECHDC1* (Hs00929453_m1); *SAT1* (spermidine/spermine N1-acetyltransferase 1; Hs00161511_m1); *ASS1* (argininosuccinate synthetase-1; Hs01597989_g1); *ATP7A* (P-type ATPase copper-transporting ATPase 1; Hs00163707_m1); *dCK* (Hs01040726_m1); ABCB1 (Hs00184500_m1); and housekeeping genes *ACTB* (β-actin; Hs99999903_m1); *GAPDH* (glyceraldehyde-3-phosphate dehydrogenase; Hs99999905_m1). *GAPDH* was selected as the reference gene. Relative expression levels (fold change) were calculated using the comparative ∆∆Ct method with DataAssist 3.01 software (Thermo Fisher Scientific, Waltham, USA).

### Determination of the protein level by Western blot

All cells were seeded in 100-mm Petri dishes (Sarstedt, Nümbrecht, Germany). After 48 h, the culture medium was removed, and cells were washed twice with phosphate-buffered saline (PBS; HIIET, PAS, Wrocław, Poland). Cells were lysed in radioimmunoprecipitation assay (RIPA) buffer supplemented with protease and phosphatase inhibitor cocktails (Sigma-Aldrich, Poznań, Poland) for 15 min at 4 °C. Lysates were centrifuged at 10,000 × *g* for 15 min at 4 °C, and the supernatants were harvested. Protein concentrations were measured using the Pierce^TM^ Coomassie Plus assay (Thermo Fisher Scientific, Warsaw, Poland), and samples were stored at -80 °C.

Equal amounts of protein (25 µg per lane) were separated by sodium dodecyl sulfate-polyacrylamide gel electrophoresis (SDS-PAGE; 4%-12%, Tris-glycine) under reducing conditions and transferred onto nitrocellulose membranes (pore size 0.45 μm; Thermo Fisher Scientific, Warsaw, Poland) using a semi-dry blotting system (Cleaver Scientific, Rugby, UK). Next, membranes were washed with Tris-buffered saline (TBS; 20 mM Tris-Base, 137 mM NaCl, pH 7.6) for 5 min at room temperature and blocked with 5% skim milk in TBS containing 0.1% Tween-20 (TBST) for 60 min. After blocking, membranes were washed three times with TBST (5 min each) and incubated overnight at 4 °C with antigen-specific rabbit IgG antibodies [anti - p21 (cyclin-dependent kinase inhibitor 1) #2947S, anti - p27 (cyclin dependent kinase inhibitor 1B) #3686S, anti - βIII-tubulin #5568S, anti - Mcl1 (induced myeloid leukemia cell differentiation protein) #94296S, anti - β-tubulin #2146S, anti - Cyclin B1 #4138S, anti - ASS1 (argininosuccinate synthetase 1) #70720S, anti - MDR1/ABCB1 (multidrug resistance protein 1/ATP binding cassette subfamily B member 1) #13342S, anti - KIF11 (kinesin family member 11) #14404; all Cell Signaling Technology, Warsaw, Poland] diluted 1:1,000 in 5% bovine serum albumin (BSA) in TBST. The next day, membranes were washed three times with TBST and incubated for 1 h at room temperature with goat anti-rabbit IgG antibodies conjugated with horseradish peroxidase (HRP; Sigma-Aldrich, Warsaw, Poland) diluted 1:1,000 in 5% BSA/TBST. After an hour incubation, membranes were washed with TBST three times for 5 min. Chemiluminescence was detected using the West Pico substrate (Thermo Fisher Scientific, Warsaw, Poland), and bands were visualized on a GelLogic 1500 imaging system (Carestream, Rochester, NY, USA).

After visualization, membranes were washed three times (10 min each) with 10 mM PBS containing 0.05% Tween (PBST, pH 7.4) and incubated for 1 h at room temperature with mouse anti-β-actin IgG antibodies (#MA5-15739; Invitrogen, Warsaw, Poland) diluted 1:4,000 in 0.5% skim milk in PBST. Following three washes with PBST, membranes were incubated for 1 h with HRP-conjugated rabbit anti-mouse IgG antibodies (#M7023, Sigma-Aldrich, Poznań, Poland) diluted 1:2,000 in 0.5% skim milk in PBST. After washing (three times, 5 min each), signals were detected as described above. Data were analyzed using ImageJ 1.8.0 and GraphPad Prism 7.03.

### Intracellular glutathione level assessment

Cells were seeded in 24-well plates (Greiner BioOne, Kremsmünster, Austria) at a density of 2 × 10^3^ cells/well. After 24 h, glutathione levels were measured using the GSH/GSSG-Glo^TM^ assay (Promega GhbH, Walldorf, Germany) according to the manufacturer’s protocol, enabling determination of both total and reduced glutathione. In parallel, experiments were conducted with cells pretreated for 24 h with 100 µM buthionine sulfoximine (BSO), an inhibitor of γ-glutamylcysteine synthetase^[13]^. Results were analyzed using GraphPad Prism 7.03.

### Statistical analysis

All experiments were performed at least in triplicate. Data were analyzed utilizing the appropriate statistical methods and software as specified under each section. Normality and variance were assessed with the Shapiro-Wilk and Brown-Forsythe tests, respectively. One-way ANOVA followed by appropriate post-hoc tests was applied where applicable. Details of the statistical tests used are provided in the corresponding tables and figures.

All figures were prepared in GraphPad Prism 7.03 software.

## RESULTS

### UBC cell lines exhibit differential drug-resistance profiles

**Figure 1:**
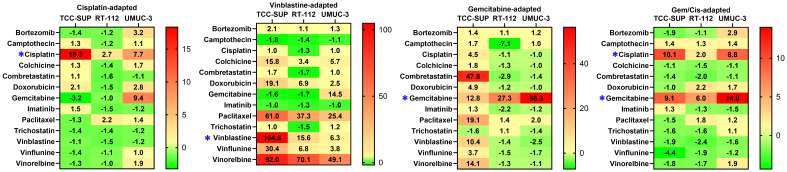
Drug RIs for all drug-adapted cell lines. * indicates the drug(s) used to generate each drug-adapted subline. RIs: Resistance indices.

A set of thirteen anticancer drugs was used to determine cell line sensitivity [Table 2]. The set comprised compounds exhibiting diverse mechanisms of action and molecular targets. Minor discrepancies in drug sensitivity were observed for parental cell lines, with the ratio between the most and the least sensitive cell line exceeding 5, recorded only for camptothecin and cisplatin. However, drug-adapted cell lines showed significant differences in resistance profiles [Figure 1]. Cisplatin exposure resulted in resistance indices (RI^CDDP^) of 18.3, 2.7, and 7.7 for TCC-SUP^CDDP^, RT-112^CDDP^, and UMUC-3^CDDP^, respectively. Only minor cross-resistance was observed with doxorubicin (Dox) in UMUC-3^CDDP^ (RI^Dox^ = 2.7) and TCC-SUP^CDDP^ (RI^Dox^ = 2.1), and with bortezomib (BZT) in UMUC-3^CDDP^ (RI^BZT^ = 3.2). Additionally, UMUC-3^CDDP^ exhibited moderate resistance to gemcitabine (RI^Gem^ = 9.4), whereas TCC-SUP^CDDP^ exhibited slightly increased sensitivity to this antimetabolite (RI^Gem^ = 0.31). Cells exposed to vinblastine developed strong resistance, with RI^VBL^ values of 104.8, 15.6, and 6.3 for TCC-SUP^VBL^, RT-112^VBL^, and UMUC-3^VBL^, respectively. Additionally, strong cross-resistance, particularly in TCC-SUP^VBL^, was observed against other antimitotic agents (colchicine, paclitaxel, vinflunine, and vinorelbine). Interestingly, no cross-resistance was detected with combretastatin A4, another antimitotic agent. All vinblastine-resistant sublines also developed moderate to strong cross-resistance to doxorubicin (maximum RI^Dox^ = 19.0 in TCC-SUP^VBL^). In addition, UMUC-3^VBL^ developed strong cross-resistance to gemcitabine (RI^Gem^ = 14.5). Notably, gemcitabine-treated sublines displayed high resistance, with RI^Gem^ values of 12.8, 27.3, and 56.3 for TCC-SUP^Gem^, RT-112^Gem^, and UMUC-3^Gem^, respectively. TCC-SUP^Gem^ additionally developed slight cross-resistance to cisplatin (RI^CDDP^ = 4.5) and doxorubicin (RI^Dox^ = 4.9, but strong cross-resistance to combretastatin A4 (CA4; RI^CA4^ = 47.8), paclitaxel (PTX; RI^PTX^ = 19.1), vinblastine (RI^VBL^ = 10.4), and vinorelbine (VRL; RI^VRL^ = 14.1). Such extensive cross-resistance was not observed in RT-112^Gem^ or UMUC-3^Gem^. RT-112^Gem^ showed significantly increased sensitivity to camptothecin (CPT; RI^CPT^ = -7.1). This phenomenon was not observed in TCC-SUP^CDDP/Gem^ (RI^CDDP^ = 10.1, RI^Gem^ = 9.1), for which even a slight increase in sensitivity toward vinflunine (VFL) was recorded (RI^VFL^ = -4.4). RT-112^CDDP/Gem^ developed only slight resistance to cisplatin (RI^CDDP^ = 2.0) and moderate resistance to gemcitabine (RI^Gem^ = 6.0), along with slight cross-resistance to doxorubicin (RI^Dox^ = 2.2). UMUC-3^CDDP/Gem^ developed moderate resistance to cisplatin (RI^CDDP^ = 8.8) and gemcitabine (RI^Gem^ = 6.0), with slight cross-resistance only to bortezomib (RI^BZT^ = 2.8).

**Table 2:** Drug-resistance profiles of UBC cell lines after 72-h exposure to compounds at various concentrations, measured by SRB assay

**IC_50_ (95%CI)**					
**Compound**	**Parental**	**CDDP**	**VBL**	**Gem**	**CDDP/Gem**
**TCC-SUP**					
Bortezomib (nM)	5.7 (5.2-6.2)	4.2 (3.8-4.6)	11.8 (11.2-12.5)	7.7 (7.4-8.1)	3.0 (2.7-3.1)
Camptothecin (nM)	43.5 (34.9-53.5)	55.3 (35.1-81.3)	24.2 (22.1-26.4)	71.8 (46.1-103.5)	59.8 (44.5-78.2)
Cisplatin (µM)	1.5 (1.2-1.8)	27.5 (24.4-31.0)	1.5 (1.3-1.7)	6.7 (6.0-7.4)	15.1 (13.4-17.4)
Colchicine (nM)	8.8 (8.1-9.6)	11.0 (9.7-12.6)	139.1 (100.3-192.6)	15.9 (13.1-19.5)	7.8 (6.9-8.6)
Combretastatin A4 (nM)	2.3 (2.1-2.6)	2.6 (2.3-2.9)	3.8 (3.2-4.6)	110.0 (99.8-123.6)	1.6 (1.4-1.9)
Doxorubicin (nM)	69.6 (52.8-89.5)	144.7 (109.4-185.6)	1,326 (1,155-1,517)	341.3 (274.9-418.3)	68.2 (49.1-90.1)
Gemcitabine (nM)	10.4 (3.4-20.6)	3.2 (1.5-4.9)	6.6 (5.8-7.5)	133.2 (73.2-205.1)	95.1 (80.4-111.5)
Imatinib (µM)	13.1 (12.3-13.9)	19.4 (18.2-20.6)	12.7 (11.8-13.7)	17.3 (16.7-17.9)	16.5 (15.4-17.7)
Paclitaxel (nM)	1.6 (1.4-1.8)	1.2 (1.1-1.4)	97.6 (89.6-106.0)	30.6 (27.6-33.9)	1.1 (1.0-1.3)
Trichostatin A (nM)	140.6 (124.5-159.4)	101.9 (90.7-114.2)	144.3 (123.5-166.3)	90.6 (60.5-120.5)	89.5 (69.4-108.0)
Vinblastine (nM)	0.25 (0.21-0.29)	0.22 (0.19-0.26)	26.2 (23.2-29.6)	2.6 (2.2-3.2)	0.13 (0.11-0.15)
Vinflunine (nM)	23.7 (20.6-27.0)	16.9 (14.1-20.1)	720.4 (636.8-820.0)	86.7 (76.2-98.4)	5.5 (4.1-7.0)
Vinorelbine (nM)	0.75 (0.67-0.84)	0.60 (0.50-0.71)	69.0 (57.7-82.0)	10.6 (8.8-12.7)	0.42 (0.36-0.49)
**RT-112**					
Bortezomib (nM)	5.7 (5.4-6.00)	4.8 (4.3-5.3)	6.0 (5.6-6.6)	6.4 (5.9-7.2)	5.3 (4.5-6.3)
Camptothecin (nM)	123.6 (83.5-172.3)	106.8 (72.3-147.6)	86.4 (58.0-122.2)	17.6 (7.7-30.5)	162.6 (101.3-238.3)
Cisplatin (µM)	9.1 (7.8-10.4)	24.2 (20.8-28.1)	7.3 (5.8-9.1)	8.3 (6.0-11.1)	17.9 (15.5-20.8)
Colchicine (nM)	21.2 (19.4-23.3)	15.4 (14.4-16.5)	72.4 (66.5-78.7)	16.7 (15.9-17.7)	14.0 (12.8-15.5)
Combretastatin A4 (nM)	7.7 (6.6-9.0)	4.9 (4.5-5.30)	4.6 (4.2-5.0)	2.7 (2.5-2.9)	3.9 (3.4-4.5)
Doxorubicin (nM)	74.6 (56.4-96.3)	50.7 (38.5-64.9)	513.7 (387.0-675.0)	60.6 (44.8-80.0)	166.0 (140.1-195.2)
Gemcitabine (nM)	11.9 (7.7-17.4)	11.6 (3.1-19.4)	7.1 (3.9-11.1)	325.0 (254.5-413.5)	70.9 (44.8-103.5)
Imatinib (µM)	13.5 (12.1-14.8)	8.9 (6.8-10.8)	10.8 (9.9-11.9)	6.2 (5.7-7.1)	10.6 (9.2-12.3)
Paclitaxel (nM)	1.2 (0.9-1.6)	2.6 (2.3-3.0)	44.8 (39.6-50.9)	1.7 (1.6-1.8)	2.1 (1.8-2.3)
Trichostatin A (nM)	112.4 (104.4-121.1)	82.6 (68.2-98.0)	76.3 (64.2-89.4)	118.8 (108.5-129.6)	70.0 (58.3-83.2)
Vinblastine (nM)	0.50 (0.43-0.58)	0.34 (0.32-0.36)	7.8 (7.1-8.6)	0.35 (0.34-0.37)	0.21 (0.19-0.22)
Vinflunine (nM)	64.4 (57.6-71.8)	56.4 (52.5-60.6)	439.6 (384.0-506.8)	43.3 (41.1-45.6)	33.4 (28.6-38.1)
Vinorelbine (nM)	0.92 (0.80-1.06)	0.90 (0.78-1.03)	64.5 (58.4-71.8)	0.74 (0.68-0.81)	0.53 (0.41-0.66)
**UMUC-3**					
Bortezomib (nM)	5.4 (4.8-6.1)	17.5 (15.3-20.0)	6.8 (6.0-7.8)	6.2 (5.5-7.0)	15.4 (12.9-18.2)
Camptothecin (nM)	17.0 (14.7-19.8)	19.0 (16.8-21.4)	15.1 (11.9-18.5)	17.0 (14.6-20.0)	23.6 (20.5-27.0)
Cisplatin (µM)	2.6 (2.2-2.89)	20.0 (17.9-22.2)	2.6 (1.8-3.4)	2.5 (1.7-3.3)	22.9 (20.1-26.0)
Colchicine (nM)	9.9 (8.4-11.7)	16.7 (13.7-20.4)	56.5 (44.5-72.2)	9.5 (7.4-11.4)	9.0 (7.7-10.2)
Combretastatin A4 (nM)	1.8 (1.4-2.1)	1.6 (1.2-1.9)	1.8 (1.5-2.1)	1.3 (1.1-1.6)	1.6 (1.3-2.0)
Doxorubicin (nM)	34.8 (27.5-42.8)	95.7 (82.4-110.1)	85.9 (62.6-112.9)	33.4 (29.6-37.6)	58.5 (45.8-72.8)
Gemcitabine (nM)	16.0 (15.3-16.7)	150.5 (123.8-181.7)	232.0 (211.2-255.2)	900.7 (753.6-1,066.0)	224.0 (199.7-250.0)
Imatinib (µM)	21.0 (17.7-24.8)	17.6 (15.4-20.2)	20.5 (17.4-24.3)	18.3 (16.1-20.8)	14.2 (12.6-16.3)
Paclitaxel (nM)	1.6 (1.1-2.1)	2.2 (1.9-2.6)	40.6 (36.1-45.9)	3.2 (2.3-4.3)	1.9 (1.5-2.3)
Trichostatin A (nM)	49.6 (38.5-63.0)	42.2 (23.1-63.6)	61.7 (50.3-74.6)	36.4 (27.8-46.1)	52.3 (40.8-65.3)
Vinblastine (nM)	0.83 (0.55-1.21)	0.72 (0.61-0.86)	5.2 (4.1-6.5)	0.33 (0.27-0.41)	0.53 (0.31-0.83)
Vinflunine (nM)	55.8 (45.7-67.8)	56.7 (39.0-96.6)	210.8 (158.9-279.1)	32.9 (24.4-44.5)	45.0 (35.0-58.4)
Vinorelbine (nM)	0.86 (0.72-1.03)	1.6 (1.4-1.8)	42.2 (30.6-59.7)	0.76 (0.60-0.97)	1.6 (0.9-2.7)

Data are presented as IC_50_ (95%CI). UBC: Urinary bladder cancer; SRB: sulforhodamine B; CDDP: cisplatin; VBL: vinblastine; Gem: gemcitabine; TCC: transitional cell carcinoma.

Overall, drug-resistance profiling across fifteen cell lines revealed significant discrepancies among sublines derived from different parental cell lines. In some cases, extended cross-resistance emerged (e.g., TCC-SUP^VBL^), whereas in others, sensitivity to secondary drugs remained almost unchanged (e.g., RT-112^CDDP/Gem^) or even increased (e.g., RT-112^Gem^ to camptothecin). These findings indicate that the tested cell lines developed drug resistance through diverse molecular mechanisms.

### UBC cell lines exhibit differential profiles of selected drug-resistance-related genes

To address discrepancies observed in drug-resistance profiling, we evaluated the expression of several genes often associated with drug-resistant phenotypes and with cancer aggressiveness, invasiveness, and poor prognosis [Table 3]. We selected a targeted set of genes to cover a broad range of potential drug resistance mechanisms. The goal was to identify markers that could distinguish between our various resistant cell lines. While this small panel cannot capture all possible resistance mechanisms - especially across three drugs with distinct modes of action - it successfully uncovered notable differences between the models.

**Table 3:** Representative resistance-related genes assessed in the current study

**Gene**	**Protein name**	**Related drug**	**Mechanism**	**Ref.**
*ABCB1*	ATP-dependent translocase	Multiple drugs	↑, drug efflux pump	UniProt no. P08183; [14]
*ASS1*	Argininosuccinate synthase	Platinum-based drugs	↓, metabolic reprogramming	UniProt no. P00966; [15-17]
*ATP7A*	Copper-transporting ATPase 1	Platinum-based drugs	Drug sequestration/efflux	UniProt no. Q04656; [18]
*dCK*	Deoxycytidine kinase	Pyrimidine antimetabolites	Mut, ↓, prevents drug activation	UniProt no. P27707; [19]
*ECHDC1*	Ethylmalonyl-CoA decarboxylase	Gemcitabine	↑, altered metabolism	UniProt no. Q9NTX5; [20]
*hENT1*	Equilibrative nucleoside transporter 1	Gemcitabine	↓; reduced drug uptake	UniProt no. Q99808; [21]
*MT2A*	Metallothionein-2	Platinum-based drugs	↓; drug inactivation	UniProt no. P02795; [22]
*SAT1*	Diamine acetyltransferase 1	Cisplatin	↓; altered polyamine metabolism	UniProt no. P21673; [17]
*SLC3A1*	Neutral and basic amino acid transport protein	Several drugs, incl. cisplatin and doxorubicin	↑, altered nutrient transport	UniProt no. Q07837; [23]
*TUBβ3*	Tubulin βIII	Tubulin-interacting agents	↑, mut, altered drug target	UniProt no. Q13509; [24]

↑: Upregulated; ↓: downregulated, mut: mutated. ATP: Adenosine triphosphate.

**Figure 2:**
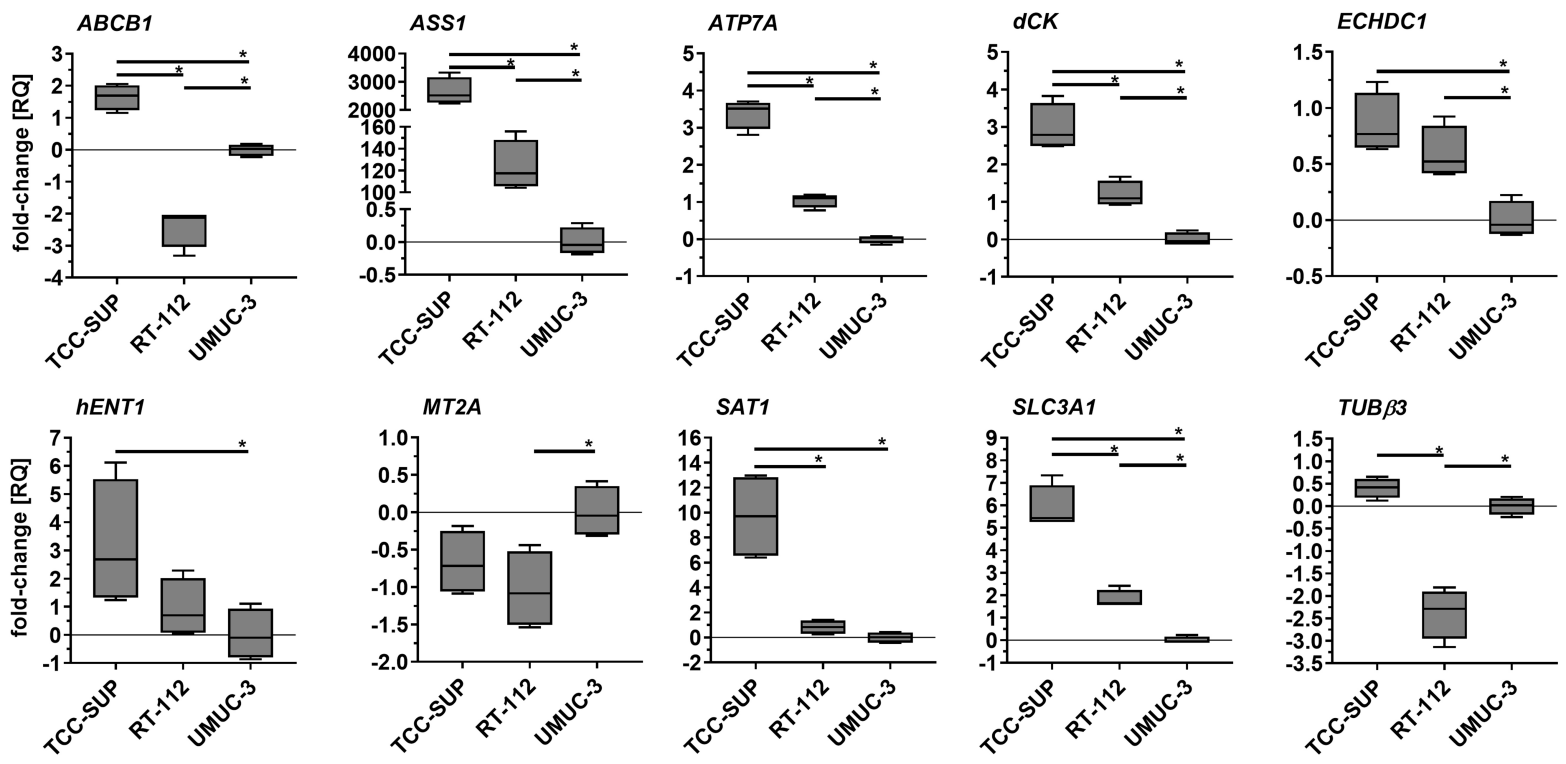
Comparison of selected gene expression in parental cell lines. Quantitative PCR results were normalized to *GAPDH* and expressed relative to the mean mRNA level of each gene in UMUC-3. Results (*n* = 4) are shown as box-and-whisker plots, with whiskers indicating minimum and maximum values. ^*^P < 0.05, one-way ANOVA with Tukey’s post-hoc test. PCR: Polymerase chain reaction; ANOVA: analysis of variance.

Parental cell lines exhibited significantly different expression profiles for most of the genes tested [Figure 2]. Statistically significant differences were observed, for example, in *ABCB1* and *TUBβ3* (with RT-112 exhibiting the lowest expression), as well as in *ATP7A*, *dCK*, *SAT1*, and *SLC3A1* (with TCC-SUP exhibiting the highest expression). Discrepancies in *ASS1* expression were especially striking: TCC-SUP and RT-112 showed 2,646- and 123-fold overexpression, respectively, compared to UMUC-3.

**Figure 3:**
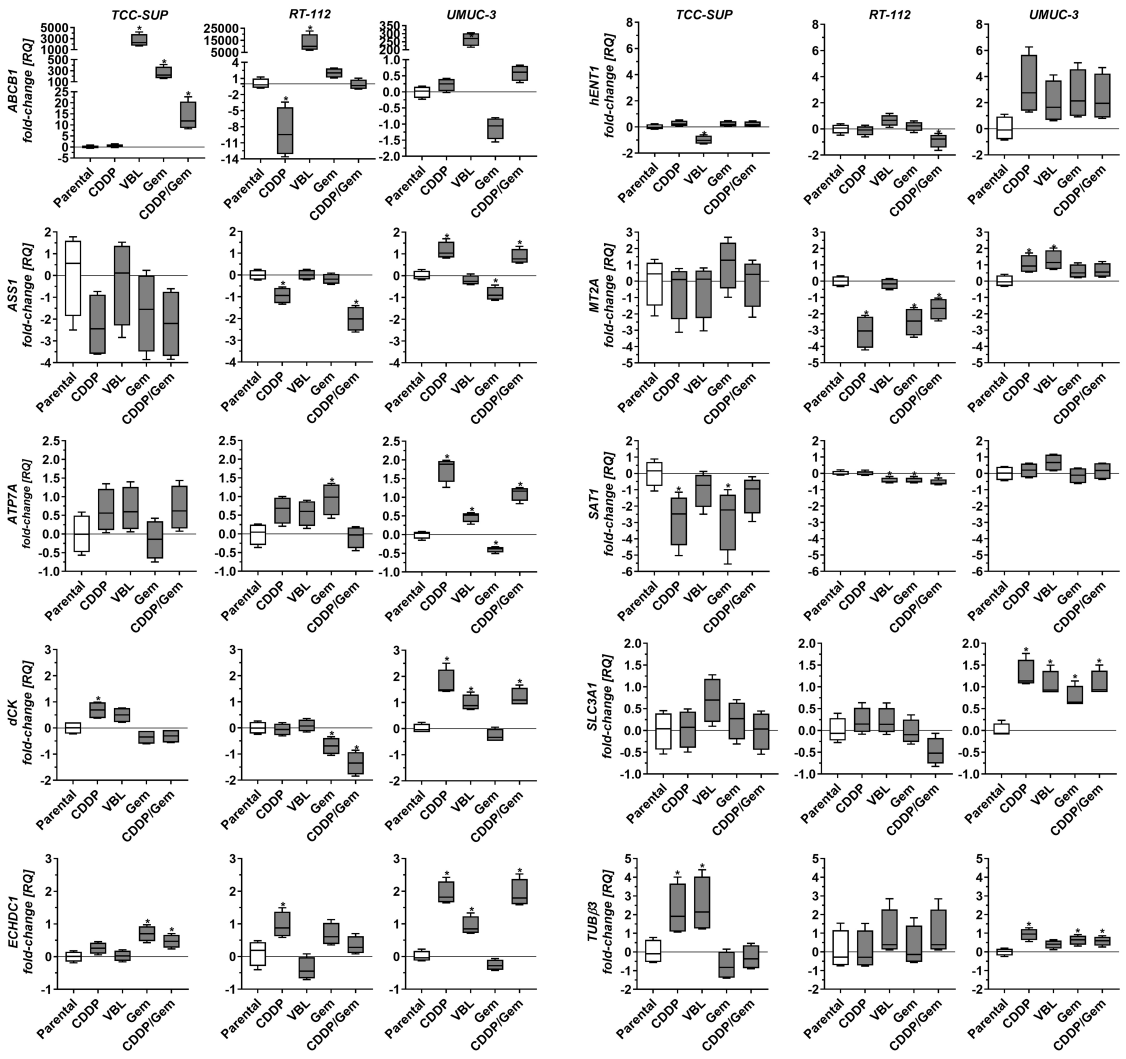
Comparison of selected gene expression in drug-resistant sublines relative to parental cell lines. Quantitative real-time PCR results were normalized to *GAPDH* and expressed relative to the mean mRNA level of each gene in the corresponding parental line. Results (*n* = 4) are shown as box-and-whisker plots, with whiskers indicating minimum and maximum values. ^*^P < 0.05 to parental line, oneway ANOVA with Dunnett’s post-hoc test. PCR: Polymerase chain reaction; ANOVA: analysis of variance.

Drug-adapted cell lines also displayed distinct expression profiles [Figure 3]. TCC-SUP^CDDP^ (the only cisplatin-adapted line in this set) showed decreased *SAT1* expression and increased *dCK* and *TUBβ3* expression. RT-112^CDDP^ exhibited reduced *ABCB1*, *ASS1*, and *MT2A* levels and increased *ECHDC1* expression. UMUC-3^CDDP^ displayed elevated *ASS1*, *ATP7A*, *MT2A*, and *SLC3A1* as well as increased *dCK*, *ECHDC1*, and *TUBβ3*. All three vinblastine-adapted cell lines exhibited significantly increased *ABCB1* expression, with the highest increases observed in RT-112^VBL^ and TCC-SUP^VBL^ (12,433- and 2,271-fold, respectively, relative to the parental lines). Because *ABCB1* expression is four times lower in RT-112 compared to TCC-SUP, the parentalcorrected *ABCB1* increase in TCC-SUP^VBL^ is nearly equivalent to that in RT-112^VBL^. Additionally, TCC-SUP^VBL^ (the only VBL-adapted line) exhibited decreased *hENT1* and increased *TUBβ3*, whereas RT-112^VBL^ displayed slightly decreased *SAT1* expression. UMUC-3^VBL^ showed increased *ATP7A*, *dCK*, *ECHDC1*, *MT2A*, and *SLC3A1*. Gemcitabine-adapted lines also showed distinct patterns. TCC-SUP^Gem^ and TCC-SUP^Gem/CDDP^ (the only gemcitabine-adapted lines in this set) exhibited increased *ABCB1* and *ECHDC1*. RT-112^Gem^ and RT-112^Gem/CDDP^ displayed decreased *dCK*, *MT2A*, and *SAT1* (similar to TCC-SUP^Gem^ for *SAT1*), with additional *ASS1* downregulation in RT-112^Gem/CDDP^ and *ATP7A* upregulation in RT-112^Gem^. UMUC-3^Gem^ and UMUC-3^Gem/CDDP^ exhibited elevated *SLC3A1* and *TUBβ3*, with variable changes in *ASS1*, *ATP7A*, *dCK*, and *ECHDC1* (both genes upregulated in the Gem/CDDP subline but downregulated or unchanged in the gemcitabine-only line).

The heterogeneous gene expression profiles observed in drug-resistant cell lines (even among lines adapted to the same drug) indicate that each line employs a different strategy to achieve a drug-resistant phenotype. This diversity is an important feature of this cell line panel when considering its use in drug resistance studies.

### UBC cell lines: drug-resistant profiles associated with significant differences in selected protein levels

To further characterize the newly derived drug-adapted cell lines, we analyzed variations in selected protein levels via Western blot followed by densitometry. We focused on the protein expression of ABCB1 and ASS1, as these genes exhibited the most variable transcript levels across the parental cell line panel. Additionally, we evaluated proteins linked to cell cycle progression (Cyclin B1, p21, p27), mitosis (KIF11, tubulins), and apoptosis regulation (Mcl-1). This targeted protein set allowed us to identify key differences between the cell lines, such as distinct p21/p27 expression profiles, and to further delineate significant variations in their drug resistance mechanisms, even with a limited number of proteins examined.

**Figure 4:**
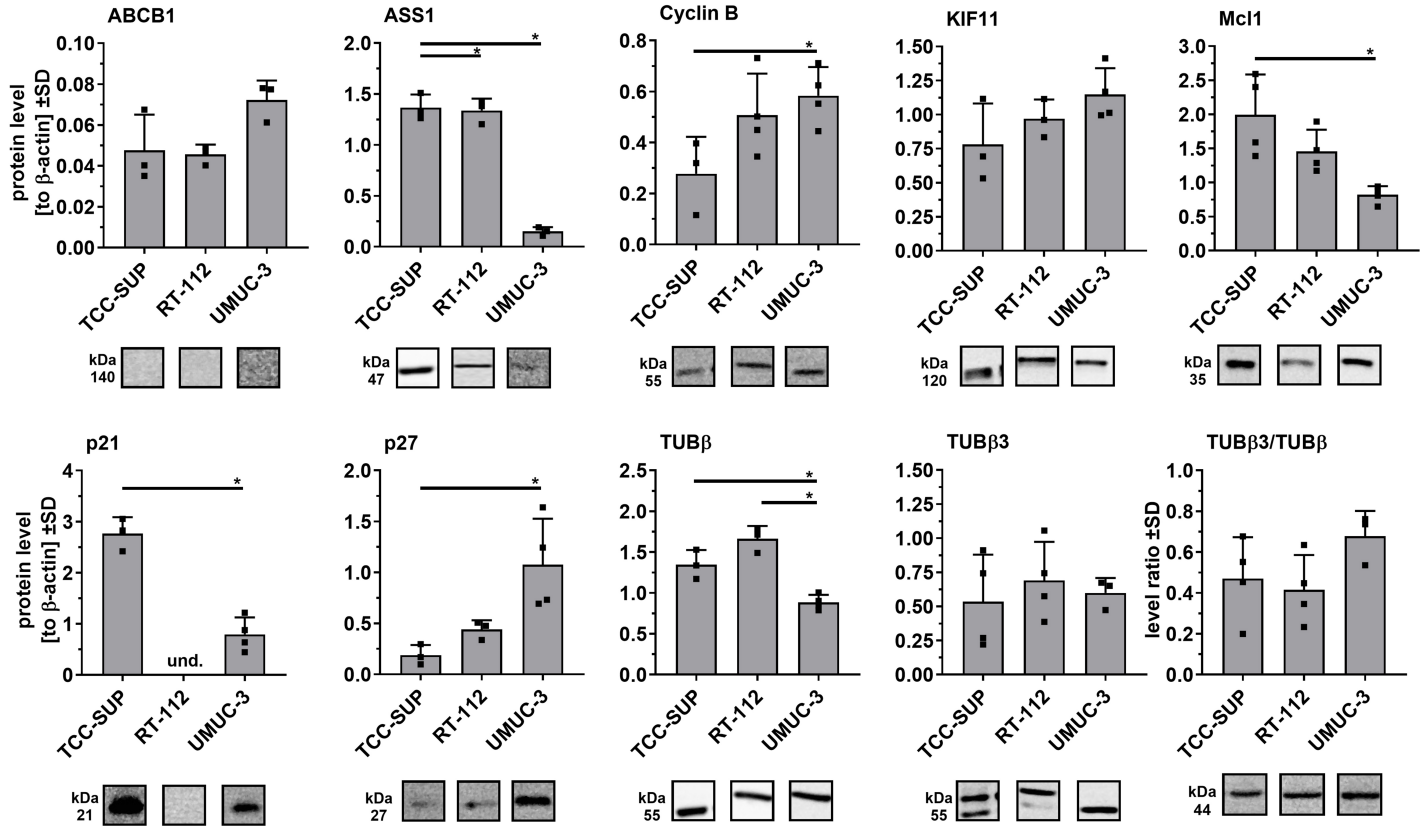
Comparison of selected protein levels in parental cell lines using Western blot analysis. Densitometric values for each protein (*n* = 3-4) were normalized to β-actin and presented as mean ± SD, with ▪ symbols indicating raw values. ^*^P < 0.05, one-way ANOVA with Tukey’s post-hoc test for multiple comparisons (except p21 results, analyzed by two-tailed *t*-test); und: undetectable. SD: Standard deviation; ANOVA: analysis of variance.

In the parental cell lines [Figure 4], significant differences were observed in ASS1, Mcl-1, Cyclin B, p21, p27, and β-tubulin, particularly between TCC-SUP and UMUC-3. Notably, p21 was not detected in RT-112 cells, consistent with the previously reported absence of functional p21 protein in this line^[25]^.

**Figure 5:**
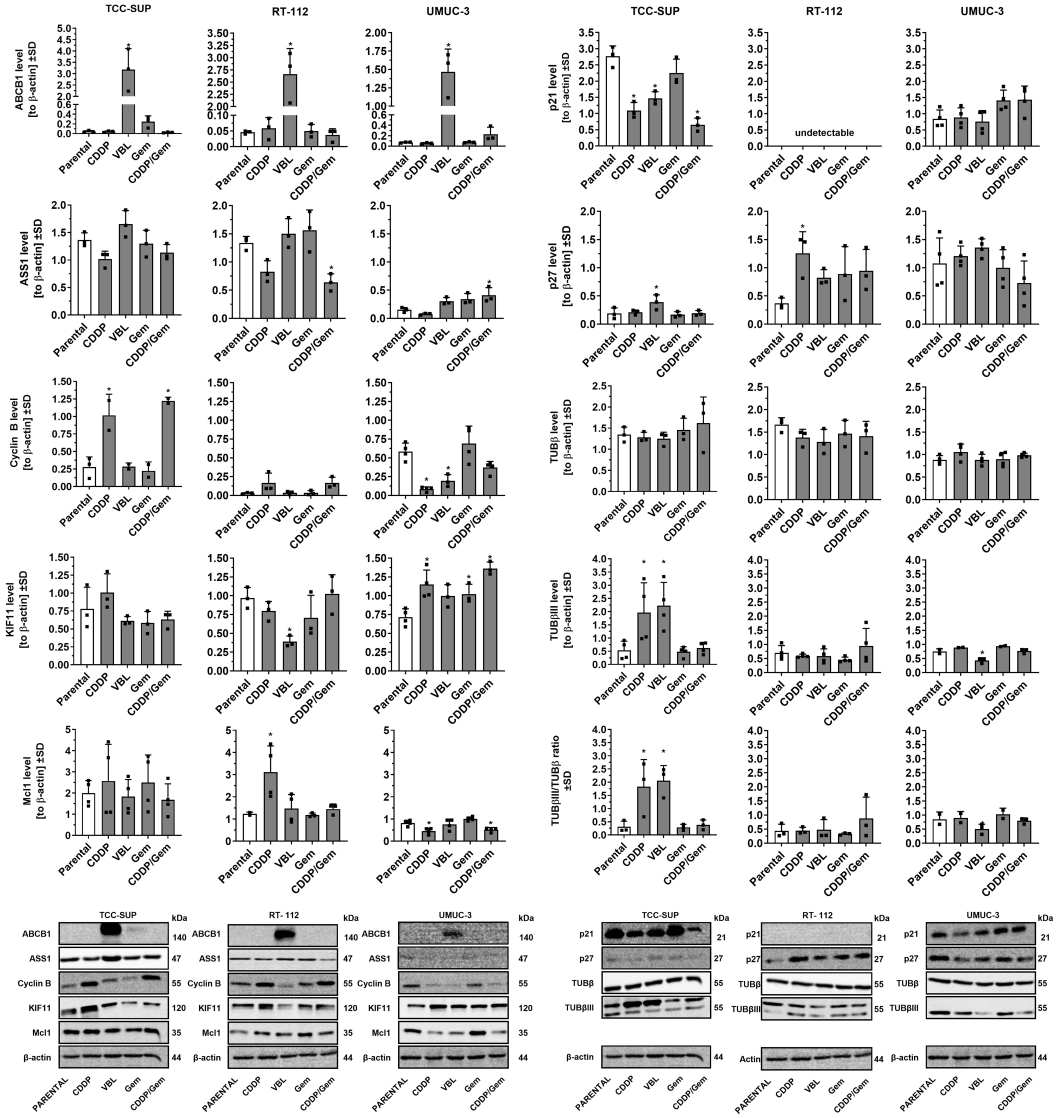
Comparison of selected protein levels in drug-resistant sublines versus parental cell lines. Densitometric values (*n* = 3-4) were normalized to β-actin and presented as mean ± SD, with ▪ symbols indicating raw values. ^*^P < 0.05, one-way ANOVA with Dunnett’s post-hoc test compared to the corresponding parental cell line; und: undetectable. SD: Standard deviation; ANOVA: analysis of variance.

Drug-adapted cell lines also showed significant differences in selected protein levels [Figure 5]. In cisplatin-adapted cell lines, marked changes were observed for Cyclin B, Mcl1, p21, and p27. In TCC-SUP^CDDP^ and RT-112^CDDP^ cells, Cyclin B and Mcl1 levels increased, whereas UMUC-3^CDDP^ displayed the opposite trend. p21 expression significantly decreased in TCC-SUP^CDDP^, remained undetectable in RT-112 sublines, and showed no significant changes in UMUC-3^CDDP^. In contrast, p27 expression increased in RT-112^CDDP^. Importantly, TCC-SUP^CDDP^ was the only cisplatin-resistant cell line with elevated β-tubulin III expression, at levels comparable to those in TCC-SUP^VBL^. All vinblastine-resistant cell lines showed strong expression of the ABCB1 efflux pump. Additionally, UMUC-3^VBL^ cells exhibited reduced Cyclin B and β-tubulin III, while TCC-SUP^VBL^ cells exhibited increased p27 and decreased p21. Continuous gemcitabine treatment had minimal effects on protein levels in any cell line. However, combined gemcitabine/cisplatin-adapted cell lines exhibited altered ASS1 expression (decreased in RT-112^CDDP/Gem^ and increased in UMUC-3^CDDP/Gem^) and a slight reduction in Mcl1 in UMUC-3^CDDP/Gem^. Notably, relative ASS1 expression was consistently lower in UMUC-3 parental cells compared with TCC-SUP and RT-112. Furthermore, UMUC-3 sublines adapted to cisplatin and/or gemcitabine showed elevated KIF11, whereas RT-112^VBL^ exhibited significantly lowered levels of this kinesin.

The distinct protein expression profiles observed in drug-adapted cell lines that originated from different parental lines indicate significant discrepancies in the metabolic pathways involved in drug resistance. These observations underscore the utility of these newly developed cell lines as valuable models for studying mechanisms of drug resistance.

### UBC drug-resistant cells present diverse total and reduced glutathione profiles

**Figure 6:**
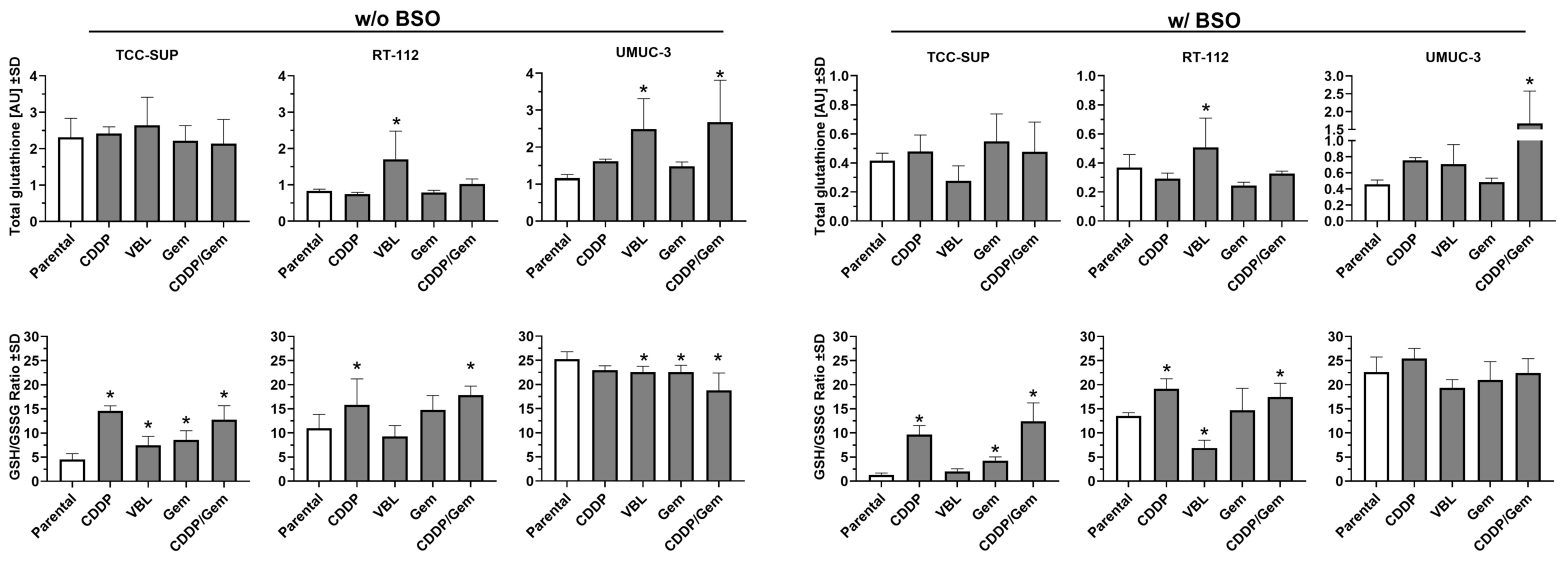
Comparison of total glutathione levels (upper panels) and GSH/GSSG ratio (lower panels) in drug-resistant sublines relative to their parental cell lines. The left panel shows results without BSO pre-treatment, while the right panels show results after 24 h pre-treatment with 100 µM BSO. Data (*n* = 3-4) were analyzed according to the manufacturer’s protocol and are presented as mean ± SD. ^*^P < 0.05, one-way ANOVA with Dunnett’s post-hoc test, compared to the corresponding parental cell line. GSH: Glutathione; GSSG: glutathione disulfide; BSO: buthionine sulfoximine; SD: standard deviation; ANOVA: analysis of variance.

Since glutathione (GSH) plays a pivotal role in phase II detoxification pathways - acting as a key regulator of cellular redox status and involved in xenobiotic inactivation - we measured glutathione levels in newly established cell lines to determine whether changes in its levels contribute to drug resistance [Figure 6, left panel]. The parental TCC-SUP cell line exhibited more than twice the total glutathione level of the other parental lines, and this level did not change substantially after drug adaptation. In the RT-112 cell line series, adaptation to vinblastine resulted in a significant increase in total glutathione level (2.0-fold change), similar to the UMUC-3^VBL^ cell line (2.2-fold change). The greatest increase was observed in the UMUC-3^CDDP/Gem^ cell line (2.3-fold change). Interestingly, the parental TCC-SUP cell line exhibited the lowest GSH/GSSG ratio among all lines tested (4.5). All of its drug-adapted sublines showed a significant increase, with the highest ratios observed in TCC-SUP^CDDP^ and TCC-SUP^CDDP/Gem^ (14.6 and 12.7, respectively). This suggests that glutathione recycling to its reduced form, rather than increased synthesis, was the predominant response to xenobiotic exposure in the culture medium. In RT-112 cells (parental ratio = 10.9), adaptation to CDDP and CDDP/Gem resulted in significant increases in the GSH/GSSG ratio (15.8 and 17.8, respectively), but the fold changes (~1.5-fold) were smaller than those in TCC-SUP sublines (~3.0-fold). In contrast, the UMUC-3 parental cell line showed the highest GSH/GSSG ratio (25.3), and three of its sublines (VBL, Gem, and CDDP/Gem) showed decreases, with the largest reduction observed in UMUC-3^CDDP/Gem^ (18.8). Nevertheless, UMUC-3 sublines exhibited the least substantial changes in the GSH/GSSG ratio compared to the other cell lines.

To assess cell line susceptibility to glutathione depletion, the experiments were repeated with cells treated with 100 µM BSO for 24 h [Figure 6, right panel]. BSO treatment markedly reduced total glutathione levels in all parental lines, with the greatest decrease observed in TCC SUP (5.6-fold change). Comparable glutathione depletion was also observed in all of its sublines. Most other cell lines exhibited at least a 2-fold decrease in total glutathione compared to the BSO-free condition. The only exception was the UMUC-3^CDDP/Gem^ subline, which showed only a 1.6-fold reduction, suggesting higher resistance to BSO-induced depletion. The GSH/GSSG ratio did not change substantially after BSO pre-treatment; thus, parental and drug-resistant cell lines maintained comparable relationships (differences did not exceed ~1.5-fold). Notable exceptions were the parental TCC-SUP cell line and its TCC-SUP^VBL^ and TCC-SUP^CDDP/Gem^ sublines, which exhibited 6.5-, 3.7- and 2.0-fold changes, respectively. These results indicate that in TCC SUP cells, glutathione depletion due to synthesis blockage is accompanied by impaired recycling of glutathione into its reduced form, potentially rendering them more susceptible to oxidative stress.

## DISCUSSION

A pivotal finding of our study is that the adaptive strategies for drug resistance are profoundly influenced by the intrinsic genetic context of the parental cancer cell. The distinct resistance profiles of the TCC-SUP, UMUC-3, and RT-112 sublines can be plausibly linked to their known driver mutations (*PIK3CA*, *KRAS*, and *FGFR3*, respectively), which prime these cells for specific survival pathways when challenged with chemotherapy.

The TCC-SUP cell line, which harbors an activating *PIK3CA* (E545K) mutation, consistently developed resistance through mechanisms involving drug efflux and cell cycle deregulation. The PI3K/AKT/mTOR pathway, constitutively activated by this mutation, is a master regulator of cell growth, proliferation, and survival^[26,27]^. Its chronic activation can promote resistance by broadly enhancing cell survival signals that counteract the pro-apoptotic effects of chemotherapy^[26]^. This may explain why TCC-SUP sublines, particularly TCC-SUP^VBL^, exhibited the most potent resistance, driven by upregulation of the *ABCB1* drug efflux pump, which conferred cross-resistance to other antimitotic agents recognized as ABCB1 substrates^[28]^. The PI3K pathway has also been linked to the transcriptional regulation of ABC transporter expression^[29]^. Furthermore, the observed decrease in p21 and increase in Cyclin B in cisplatin- and vinblastine-adapted TCC-SUP cells suggest a strategy of overriding cell cycle checkpoints. Reduced p21 levels may enable cell cycle progression despite the presence of damaged DNA, thereby contributing to genomic instability^[30,31]^. Overactivation of the PI3K/AKT pathway can phosphorylate and inactivate cell cycle inhibitors, driving proliferation even in the presence of DNA damage, a hallmark of a robust resistance phenotype^[32]^. This phenotype is further supported by adaptations in glutathione metabolism; rather than increasing total glutathione, TCC-SUP sublines enhanced glutathione recycling, as indicated by a significant increase in the GSH/GSSG ratio, thereby boosting their detoxification capacity^[33,34]^.

The UMUC-3 cell line, carrying an activating *KRAS* (G12C) mutation, demonstrated a preference for metabolic adaptations, particularly those involving GSH. Oncogenic *KRAS* is well-documented to rewire cellular metabolism to meet the high anabolic and redox demands of cancer cells^[35]^. *KRAS* mutations drive upregulation of glycolysis and glutamine utilization, supplying precursors for proliferation but also increasing the production of reactive oxygen species (ROS) as a byproduct. To survive this self-induced oxidative stress, *KRAS*-mutant cells become highly dependent on antioxidant systems, particularly the glutathione pathway, which neutralizes ROS^[36,37]^. Our finding that UMUC-3 sublines consistently modulated their GSH levels and GSH/GSSG ratio aligns with this paradigm. Their reliance on glutathione was further evidenced by adaptations such as increased total glutathione levels and overexpression of the cysteine transporter SLC3A1, a key contributor to GSH synthesis^[38,39]^. For these cells, strengthening the glutathione system is a pre-existing survival mechanism that can be readily exploited to detoxify xenobiotics such as cisplatin, explaining their reliance on this pathway rather than ABCB1 upregulation.

The RT-112 cell line, characterized by an *FGFR3-TACC3* fusion, presents a more nuanced case. Activating *FGFR3* mutations are common in non-muscle-invasive bladder cancer and are typically associated with a distinct, less aggressive tumor biology^[8,40]^. The *FGFR3* signaling pathway primarily influences cell proliferation and differentiation via the RAS-MAPK and PI3K-AKT axes^[41,42]^. Unlike the broad, systemic effects of *KRAS* or *PIK3CA* mutations, the consequences of FGFR3 activation are more specific. In our study, RT-112 sublines developed resistance through multiple mechanisms without a single dominant strategy as seen in TCC-SUP or UMUC-3. For instance, resistance to vinblastine involved *ABCB1* upregulation, though to a lesser extent than in TCC-SUP. In parallel, these cells also employed a distinct glutathione-based strategy, characterized by increased glutathione synthesis rather than enhanced recycling, demonstrating metabolic flexibility^[43]^. Notably, studies have shown that resistance to FGFR inhibitors in FGFR3-driven cells can arise from the activation of bypass signaling tracks or the acquisition of new mutations, such as in *HRAS* or PI3K^[41,44]^. This suggests that FGFR3-driven networks provide cellular plasticity, allowing for the emergence of various smaller-effect resistance mechanisms rather than reliance on a single dominant one. The increased p27 levels observed in RT-112^CDDP^ may represent one such specific adaptation, inducing cell cycle arrest that permits DNA repair and survival - a strategy not observed in the other cell lines. While p27 is a cyclin-dependent kinase inhibitor, its upregulation can act as a double-edged sword. In this context, however, p27-induced cell cycle arrest may prevent drug-induced apoptosis by providing additional time for DNA repair, ultimately enhancing cell survival^[45]^.

While this study provides valuable insights into the molecular underpinnings of chemoresistance in UBC, its conclusions must be considered in the context of two primary limitations: the finite genetic scope of the cellular models and the vast complexity of drug resistance mechanisms. First, our investigation was based on three distinct cell lines - TCC-SUP, RT-112, and UMUC-3 - which were deliberately selected to represent a spectrum of disease subtypes with *PIK3CA*, *FGFR3*, or *KRAS* mutations and varying degrees of genomic instability^[8,46]^. This diversity was critical, as it revealed cell line-specific resistance strategies, such as the dichotomy in p21 function, which appeared to promote unchecked proliferation in TCC-SUP sublines but may act as an anti-apoptotic shield in UMUC-3^[47]^. Nevertheless, it is self-evident that three cell lines cannot fully recapitulate the heterogeneity of available cancer cell lines^[8]^, nor the extensive intertumoral heterogeneity observed across the entire patient population^[48,49]^. The unique genetic background of each line is likely a key determinant of the divergent resistance pathways we observed, underscoring the need for broader cell line panels to more accurately model the full clinical landscape.

Second, the therapeutic agents investigated - cisplatin, vinblastine, and gemcitabine - are known to trigger diverse resistance mechanisms, many of which were beyond the scope of our targeted analysis. For instance, our investigation into cisplatin resistance focused on ATP7A, metabolic enzymes such as ASS1 and SAT1, and metallothionein-2 (MT2A)^[17,50-53]^. However, we did not evaluate contributions from other critical pathways, including nucleotide excision repair (NER) via ERCC1, the roles of MLH1 and POLH in DNA damage tolerance, or the influence of autophagy^[13,33]^. Similarly, while our analysis of vinblastine resistance highlighted the central role of the ABCB1 efflux pump and β3-tubulin overexpression^[28,54]^, we only touched upon the distinct glutathione-based detoxification pathways that appear to differ between cell lines^[43]^. Gemcitabine resistance further highlighted this limitation: although we analyzed key mediators such as *hENT1*, *dCK*, and *ECHDC1*, the most resistant cell line, UMUC-3^Gem^, showed no significant changes in these genes, pointing toward an entirely alternative and uninvestigated resistance mechanism. One particularly intriguing possibility, not covered in our current study, is the involvement of cytidine deaminase (CDA), which converts the gemcitabine intermediate, 2′,2′-difluorodeoxycytidine (dFdC), into its inactive metabolite, 2′,2′-difluorodeoxyuridine (dFdU), along with other proteins associated with gemcitabine metabolism^[55,56]^.

Despite these limitations, our characterization provides useful landmarks for further research. By demonstrating that specific resistance mechanisms (e.g., p21/p27 modulation, ATP7A upregulation, or β3-tubulin expression) are highly dependent on cellular context, our findings make it easier to select the most appropriate sensitive/resistant cell line pairs for hypothesis-driven studies. To build on this foundation and further delineate the underlying networks, future studies with these selected cell line pairs should employ unbiased, large-scale approaches. Comprehensive transcriptomic and proteomic analyses, in particular, would be invaluable for mapping the full range of molecular adaptations driving resistance, thereby uncovering novel therapeutic targets and strategies to overcome them.

In summary, our findings strongly suggest that a cell’s foundational oncogenic drivers dictate its subsequent evolutionary path toward chemoresistance. This provides a molecular rationale for the heterogeneity of clinical responses and underscores the importance of genomic profiling, not only for selecting initial targeted therapies but also for anticipating and counteracting acquired resistance mechanisms.

## DECLARATIONS

### Availability of data and materials

Cell lines from this study are available for sharing upon reasonable request to Michaelis M (M.Michaelis@kent.ac.uk) or Cinatl J Jr. (j.cinatl@kinderkrebsstiftung-frankfurt.de). Data are contained within the article.

### Copyright

© The Author(s) 2025.
